# Bacterial profile, drug resistance pattern, clinical and laboratory predictors of ascites infection in cirrhosis patients

**DOI:** 10.1186/s12879-024-09418-6

**Published:** 2024-05-26

**Authors:** Abubeker Shemsu Helil, Shambel Araya Haile, Yohannis Birhanu, Hailemichael Desalegn, Daniel Melese Desalegn, Rozina Ambachew Geremew, Zenebe Gebreyohannes, Awad Mohammed, Daniel Dejene Wondimagegnehu, Gonfa Ayana, Anteneh Mehari Tizazu, Kassu Desta

**Affiliations:** 1 Department of Medical Laboratory Science, College of Health Science, Addis Ababa University, Addis Ababa, Ethiopia; 2 Department of Gastroenterology and Hepatology, College of Health Science, Addis Ababa University, Addis Ababa, Ethiopia; 3 Department of Gastroenterology and Hepatology, School of Medicine, St. Paul’s Hospital Millennium Medical College, Addis Ababa, Ethiopia; 4 Regional laboratory capacity building, Ethiopian Public Health Institute, Addis Ababa, Ethiopia; 5 Department of Microbiology, Immunology and Parasitology, School of Medicine, St. Paul’s Hospital Millennium Medical College, Addis Ababa, Ethiopia; 6 Department of Molecular and Translational Science, Monash University, Melbourne, Australia

**Keywords:** Ascites infection, Bacterila infection, Cirrhosis, Liver disease, Drug resistance, SBP, SAFI

## Abstract

Ascites is a pathological collection of free fluid in the peritoneal cavity, which is a common complication in patients with cirrhosis, an advanced liver disease. Bacterial infection increases the mortality rate of hospitalized patients with cirrhosis, irrespective of the severity of the liver disease. Around 60% of patients with compensated cirrhosis developed ascites within 10 years during the course of their disease. The in-hospital mortality rate due to spontaneous bacterial peritonitis (SBP) could exceed 90%, but with early diagnosis and prompt antibiotic therapy, this rate has been shown to decrease to 20%. Here, we enrolled adult (age *≥* 18) patients with liver disease with evidence of cirrhosis who developed ascites and assessed the presence of spontaneous ascites fluid infection (SAFI) in these patients. Of the total 218 patients, 22.9% (50/218) develop ascites infection. The liver organ function tests like alanine aminotransferase, aspartate aminotransferase, total bilirubin, and direct bilirubin were found to be significantly (*P* < 0.05) higher in patients with ascites fluid infection compared to patients with non-ascites fluid infection. Of the gram-negative bacteria, *K. pneumonia and E. coli* were isolated and found to be 100% resistant to amoxicillin and clavulanate. From the gram-positive bacterial isolates, *S. aureus* was only resistant to penicillin, whereas *Str. viridans* was resistant to ceftriaxone, cefotaxime, cefepime, and penicillin. On the other hand, clinical features such as a history of jaundice, low arterial blood pressure, and ultrasound results such as a shrunken liver and enlarged spleen were also independent predictors of spontaneous bacterial peritonitis. In conclusion, given the high probability of death following SAFI, early detection, and treatment, as well as knowledge of the microbial agent, resistance profile, and predictive markers in various contexts, are essential for the timely diagnosis and management of SAFI in these patients.

**Supplementary Information:**

The online version contains supplementary material available at 10.1186/s12879-024-09418-6.

## Background

Ascites is a pathological collection of free fluid in the peritoneal cavity, which is a common complication in patients with cirrhosis, an advanced liver disease [1]. Deregulation of the gut-liver immune axis in patients with cirrhosis puts them at risk of developing infection. Bacterial infection increases the mortality rate of hospitalized patients with cirrhosis, irrespective of the severity of the liver disease [2]. Bacterial infections, including spontaneous bacterial peritonitis (SBP), are common in patients with ascites and cirrhosis, and they are associated with increased morbidity and mortality. In these patients, infectious bacterial pathogens frequently arise from the commensal intestinal microbiota [3]. The most common forms of ascitic fluid infection are SBP and culture-negative neutrocytic ascites (CNNA); the other forms include secondary bacterial peritonitis, monomicrobial non-neutrocytic bacterascites (MNB), and polymicrobial bacterascites [4].

Around 60% of patients with compensated cirrhosis develop ascites within 10 years of the disease. Alongside physical examination, ultrasound evaluation and ascites fluid analysis help to rule out ascites cases caused by other than cirrhosis. Furthermore, an ascites fluid polymorphonuclear neutrophil (PMN) count greater than 250/mm3 is used to diagnose the presence of SBP in these patients [5]. A systematic analysis of the global burden of SBP among cirrhotic liver patients revealed a prevalence of 17.12%, of which the prevalence in Africa was the highest at 68.5%, whereas the prevalence in North America was the lowest at 10.81%. Similarly, the level of drug resistance among isolated pathogens was also high, at 11.77% [6]. Other studies have also shown that the prevalence of SBP is high, around 25% among patients with cirrhotic ascites [7].

It has been demonstrated that early diagnosis and fast antibiotic therapy can reduce the in-hospital death rate from over 90–20%, highlighting the criticality of early intervention [8]. Since the Enterobacterales group of bacteria is the common cause of SBP, third-generation cephalosporins (TGC) are utilized as an empirical antibiotic treatment. The emergency and spread of multidrug-resistant (MDR) and TGC-resistant organisms, as well as the increased incidence of ascites infection due to gram-positive bacteria, have created concern about the effective treatment of patients [9]. Thus, the local epidemiological pattern of microbial resistance should be considered while treating SBP patients empirically [10]. Furthermore, ruling out the source of infection (nosocomial vs. community-acquired) also has implications for the treatment of SBP infection in patients with cirrhosis [11]. This implies that in order to assist direct the practical therapy of ascites SBP in patients with hepatic cirrhosis, a continuous evaluation of common bacterial infections and their antibiograms is necessary.Clinical and laboratory parameters, as well as the bacteriological profile of ascites infection among cirrhosis patients, are lacking in Ethiopia. Here, we enrolled cirrhosis patients with ascites and assessed the presence of ascites fluid infection, the causative bacterial pathogens, and the drug susceptibility pattern of the organisms. Furthermore, we assessed different clinical, imaging, and laboratory test parameters in predicting ascites fluid infection in these patients.

## Materials and methods

A hospital-based cross-sectional study was conducted at St. Paul’s Hospital Millennium Medical College (SPHMMC) and Yekatit 12 Hospital Medical College in Addis Ababa, participants were selected through purposive sampling method.

All patients attending the internal medicine department as outpatients and inpatients in the selected hospitals for chronic liver disease with ascites comprised the source population.

Inclusion and Exclusion Criteria: Patients aged 18 and above with clinical and ultrasound evidence of cirrhosis and ascites were included in the study while study subjects with evidence of secondary peritonitis were excluded from the study.

Study Variables: The study assessed the prevalence of Spontaneous Bacterial Peritonitis (SBP), bacterial isolates from ascitic fluid culture, and the antimicrobial sensitivity pattern of bacterial isolates were considered as dependent variables. Moreover, Socio-demographic information (e.g., age and sex), clinical features (e.g., arterial blood pressure, Body Mass Index [BMI], body temperature, history of upper gastrointestinal bleeding), and laboratory features (e.g., liver function tests [LFT], complete blood count [CBC], prothrombin time/international normalized ratio [PT/INR], and hepatitis serology) served as independent variables in the study.

### Measurement and data Collection

#### Sample size determination

As far as our knowledge goes there is no previous study conducted on the prevalence of SBP among adult cirrhosis patients in Ethiopia. Therefore, we used a study done at Korle-Bu Teaching Hospital in Ghana that reported the Patterns of Ascites fluid infection among adult patients with ascites to be 21.4% to calculate our sample size.

Z = Standard score corresponding to 95% confidence level.

d = the margin of error (precision) = 5%.

n = the required sample size.

1 $$n = {\left( {Z\alpha /2} \right)^2}P\left( {1 - P} \right)/{d^2}$$

2 $$n = \left( {\left( {1.96} \right)2x0.214\left( {1 - 0.214} \right)} \right)/{\left( {0.05} \right)^2} = 258$$

About 85% of the determined sample size was achieved for this research.

#### Sampling method

The study sites were selected by purposive sampling method. A convenient sampling method was used to select each study subject. Cirrhosis patients with ascites whether symptomatic or not for SBP who presented to the internal medicine department, particularly the Gastroenterology and Hepatology unit were consecutively recruited.

### Data collection procedure

Patients’ medical records were meticulously reviewed to gather relevant history, including alcohol use, and physical characteristics indicative of liver cirrhosis. Clinical features such as ascites, hepatomegaly, splenomegaly, abdominal pain, and the presence of collateral veins were examined. Ascites diagnosis was based on specific criteria, including abdominal distention, the presence of shifting dullness, positive fluid thrill, and confirmation through diagnostic paracentesis or abdominal ultrasound scan [12–14].

#### Patient recruitment and questionnaire

After explaining the objective of the study to patients, those providing informed consent were recruited. A questionnaire covering socio-demographic data and the clinical history of patients was administered. The questionnaire encompassed information on socio-demographics, clinical signs and symptoms, laboratory investigations, ultrasound results, and other relevant aspects related to the study.

### Laboratory analysis

#### Ascitic fluid sample collection

Abdominal paracentesis was performed with an aseptic technique at the right or left iliac fossa, 3 cm above and 3 cm medial to the anterior superior iliac spine. Exactly 20 ml of ascites fluid was collected using a sterile syringe by a senior gastroenterologist or resident doctor. Subsequently, 10 ml was inoculated into a blood culture bottle at the bedside. Additionally, ascitic fluid analysis, including cell count, differential, ascitic fluid albumin, and total protein, was conducted as part of clinical utility, with data obtained from the patient card.

### Isolation of bacterial pathogen

After the sample was inoculated into a blood culture bottle (broth) (BHI and TSB), the culture medium was incubated at 37^o^C for 24 h using an incubator. After 24 h the culture medium was observed for possible microbial growth. For those who show microbial growth, a portion of the sample was transferred to a blood agar plate, chocolate agar plate, and MacConckey Agar. Mannitol salt agar was also used to isolate staphylococcus species.

### Identification of bacterial pathogen

Bacterial identification was made using biochemical tests, including, indole, citrate, oxidase, H_2_S production, lysine decarboxylase, lactose fermentation, urea hydrolysis, gas production, catalase, and mannitol fermentation from the pre-collected and stored samples.

### Antimicrobial sensitivity testing (AST)

Whenever growth was detected on the culture medium, antimicrobial sensitivity testing was done based on the identified bacterial pathogen for antibiotic disc choice. Antimicrobial sensitivity of the bacterial isolates was done by the Kirby-Bauer disc diffusion method. In the procedure, fresh sub-cultures of bacterial isolates were used after overnight growth on Muller Hinton Agar. The inoculums were prepared by suspending several of the colonies in sterile phosphate-buffered saline (pH 7.2) to achieve a turbidity of 0.5 McFarland standards. This resulted in a suspension containing approximately 1–2 × 10^8^ CFU/ml. A sterile cotton swab was dipped into the bacterial suspension, elevated above the liquid, and rotated several times against the inside wall of the tube to remove excess of the inoculum. The swabs were then streaked evenly in three different directions onto the Muller Hinton Agar. Susceptibility Testing was done by discs of choice using the Kirby-Baur disk diffusion method and the interpretation of results was made following the CLSI’s guidelines, January 2020 (30th Edition) for Sensitive, Intermediate, and Resistance Zones. Throughout the experiment Pre-analytical, analytical and post-analytical qualities were maintained. All of the results were collected using the appropriate data collection sheet.

### Ultrasound scan

All patients underwent an abdominal ultrasound scan after overnight fasting and the following details were obtained from patients’ cards: maximum vertical span of the liver; nodularity of liver surface; spleen size (length of its longest axis); presence of collateral vessels, portal vein dimension and presence of ascites.

### Blood analysis and serology

Normally all cirrhosis patients undergoing laboratory investigation for hemoglobin (HB), white blood cell count (WBC), platelet (PLT) count, international normalized ratio (INR), and serum concentrations of total protein (TP) and direct bilirubin (DB), total bilirubin (TB), serum total protein, albumin, alanine aminotransferase (ALT) and aspartate aminotransferase (AST). Alkaline phosphatase (ALP), serum sodium (Na+), potassium (K+), urea, and creatinine as well as testing for hepatitis B surface antigen (HBsAg) and antibodies to hepatitis C virus (anti-HCV-Ab) as part of clinical utility and other data were collected from patient’s cards.

### Data analysis and interpretation

Descriptive statistics were performed for all continuous variables and data was presented in appropriate graphs and tables. The prevalence of spontaneous bacteria peritonitis was determined. Further analysis was done to determine if there were any associations between spontaneous bacterial peritonitis and the clinical or laboratory test parameters. The chi-square test was used to determine the level of association. Binomial and multinomial logistic regression analyses were conducted using SPSS version 23 for possible association.The multicollinearity between the independent variables was checked using a Variance Inflation Factor (VIF), and a VIF of less than 3 was used for logistic regression. P value ˂ 0.05 was taken as a significant association for clinical or laboratory.

.

### Operational definitions

#### Cirrhosis patients

Patients with liver cirrhosis, diagnosis established by using clinical, biological, and imagistic criteria.

#### Classical spontaneous bacterial peritonitis

is defined as ascitic fluid polymorph nuclear count ≥ 250/mm3 and positive ascitic fluid culture.

#### Culture-negative neutrocytic ascites (CNNA)

are defined as ascitic fluid polymorph nuclear count ≥ 250/mm3 and negative ascitic fluid culture.

#### Non-neutrocytic bacterascites (NNBA)

is defined as ascitic fluid neutrophil count ≤ 250/mm3 with positive ascitic fluid culture.

## Results

### Socio-demographic characteristics and clinical features

A total of 218 cirrhosis patients with ascites were recruited for this study with a mean age of 38.67 ± 12.0 years (age range 19 to 76 years), with the majority of the age group between 18 and 40 years. Of the total patients, 145 (67%) were males, with a male-to-female ratio of 2.03:1. Whereas 135 (62.8%) were in the 18 years–40 years age group and 56 (25.7%) participants were single (Table 1). The clinical presentation of patients showed that 64 (29.4%) presented with upper GI bleeding, 110 (50.5%) with abdominal pain, 69 (31.7%) with jaundice, 86 (39.4%) with sleeping disturbance, 91 (41.75%) with pedal edema, 92 (42.2%) with fever, and 84 (38.5%) with chills (Table 1).

**Table 1:** Socio-demographic and clinical features of cirrhosis patients with ascites from SPSHMMC and Yekatit 12 hospital medical college, Addis Ababa, Ethiopia March 2020 to March 2021

Variables	Number	Percent
**Socio-demographic data**		
Age		
18–40	136	62.4
41–60	66	30.3
41–80	16	7.3
Sex		
Male	146	67
Female	72	33
Marital status		
Single	56	25.7
Married	158	72.5
Separated	4	1.8
**Clinical presentation**		
History of upper GI bleeding		
Yes	64	29.4
No	154	70.6
Abdominal pain		
Yes	110	50.5
No	108	49.5
History of jaundice		
Yes	69	31.7
No	149	68.3
Sleeping disturbance/memory impairment		
Yes	86	39.4
No	132	60.6
Abdominal distention		
Yes	110	50.5
No	108	49.5
Previous episode of SBP		
Yes	60	27.1
No	158	72.5
Fever		
Yes	92	42.2
No	126	57.8
Chills		
Yes	84	38.5
No	134	61.5
Pedal edema		
Yes	91	41.7
No	127	58.3
Systolic BP		
Low (˂90)	37	17
High (˃129)	28	12.8
Normal (90–129)	152	69.7
Diastolic BP		
Low (˂60)	37	17
Normal (60–90)	181	83
High (˃90)	0	0

### Cirrhosis patients with ascitic fluid infection show different organ function tests

Of the total 218 patients, 22.9% (50/218) develop ascitic infection. Organ function tests are crucial in monitoring cirrhotic patients, for instance, renal dysfunction has been identified as a robust predictor of mortality in cirrhotic patients with SBP [15]. Similarly, in our case, the liver organ function tests showed a significant difference between patients with ascitic fluid infections and those without. Thus, the median levels of alanine aminotransferase (ALT), aspartate aminotransferase (AST), total bilirubin, and direct bilirubin were found to be 37 U/L (24.1 U/L – 63.1 U/L), 53 U/L (23.9 U/L – 87.7 U/L). 1.41 mg/dL (0.625 mg/dL − 2.53 mg/dL) and 0.58 mg/dL (0.2 mg/dL − 1.62 mg/dL) respectively, which were significantly higher (*P* < 0.05) compared to patients without ascitic fluid infections, which was 25.2 U/L (17.9 U/L − 36.6 U/L), 33.5 U/L (21.1 U/L − 60 U/L), 0.67 mg/dL (0.45 mg/dL − 1.09 mg/dL) and 0.2 mg/dL (0.12 mg/dL − 0.35 mg/dL) respectively (Table 2). Likewise, a significantly (*P* < 0.05) higher level of WBC count and a significantly (*P* < 0.05) lower level of Hgb concentration were observed in patients with ascites fluid infection (Table 2). However, we observed no significant (*P* > 0.05) difference in the levels of alkaline phosphatase, urea, creatinine, and the ion concentrations of chloride, sodium, and potassium between the two groups (Table 2). Previous reports have shown that simple laboratory parameters were able to predict different chronic and non-chronic disease [16, 17] which indicate the relevance of these parameters in identifying infection among cirrhosis patients.

**Table 2:** Descriptive Statistics of laboratory parameters for patients with ascites fluid infection vs. non-ascites fluid infection at SPSHMMC and Yekatit 12 hospital medical college, Addis Ababa, Ethiopia March 2020 to March 2021(*n* = 51)

Laboratory parameters	Ascites fluid infection (*n* = 50) X(75% IQR)	Non-ascites fluid infection (*n* = 166) X(75% IQR)	*P*-Value
WBC( x 10^3^cells/mm^3^)	12.25 (9.3–14.25)	5 (3.8–7)	2.20E-16
Hgb (g/dL)	13.15 (11.7–14.5)	14.1 (12.9–15.6)	0.02662
Alanine aminotransferase (U/L)	37 (24.1–63.1)	25.2 (17.9–36.6)	0.0006311
Alkaline phosphatase (U/L)	130.5 (101–155.7)	98 (70–170)	0.08479
Aspartate amino transferase (U/L)	53 (23.9–87.7)	33.5 (21.1–60)	0.02404
Total bilirubin (mg/dL)	1.41 (0.625–2.53)	0.67 (0.45–1.09)	0.0005362
Direct Bilirubin (mg/dL)	0.58 (0.2–1.62)	0.2 (0.12–0.35)	0.00002742
Urea (mg/dL)	22.1 (17–34.9)	18.55 (15.1–28.6)	0.08542
Creatinine (mg/dL)	0.77 (0.61–1)	0.78 (0.62–1.023)	0.8906
Chloride (mmol/L)	100.7 (98.2–106)	100 (97–102.7)	0.3697
Potassium (mmol/L)	4.3 (3.7–4.7)	4.16 (3.9–4.54)	0.7924
Sodium (mmol/L)	135 (131.5–139.5)	137 (132–139)	0.5013

### Ascitic fluid analysis in cirrhosis patients show differences in protein levels

The fluid analysis for chronic liver disease patients (*n* = 218) demonstrated that 98% (50/51) of patients with ascitic fluid had an ascitic fluid neutrophil counts greater than 250 and 96.1% (49/51) of patients with SAFI had an ascites fluid albumin levels less than 0.5 g/dL. The ascitic fluid total protein showed an increased levels in non-infected individuals compared to patients with ascites infection (Table 3).

**Table 3:** Ascites fluid analysis in cirrhosis patient

Parameters	Non- SAFI		SAFI	
	Number	Percent	Number	Percent
**Ascites Fluid Neutrophil Count**				
Neutrocytic (˃250) cells/mm3)	0	0	50	98
Non-neutrocytic (˂250cells/mm3)	166	100	1	2
**Ascites Fluid ALB**				
≤ 0.5 g/Dl	14	8.4	49	96.1
0.51–0.75 g/dL	128	76.6	0	0
0.76–1.0 g/dL	18	10.8	0	0
≥ 1.1	7	4.2	2	3.9
**Ascites Fluid TP**				
≤ 1.5 g/dL	88	52.4	33	64.7
˃ 1.5 g/dL	78	46.4	18	35.3

### Culture-negative neurocytic ascites and HBV were common in cirrhosis liver patients

**Fig. 1:**
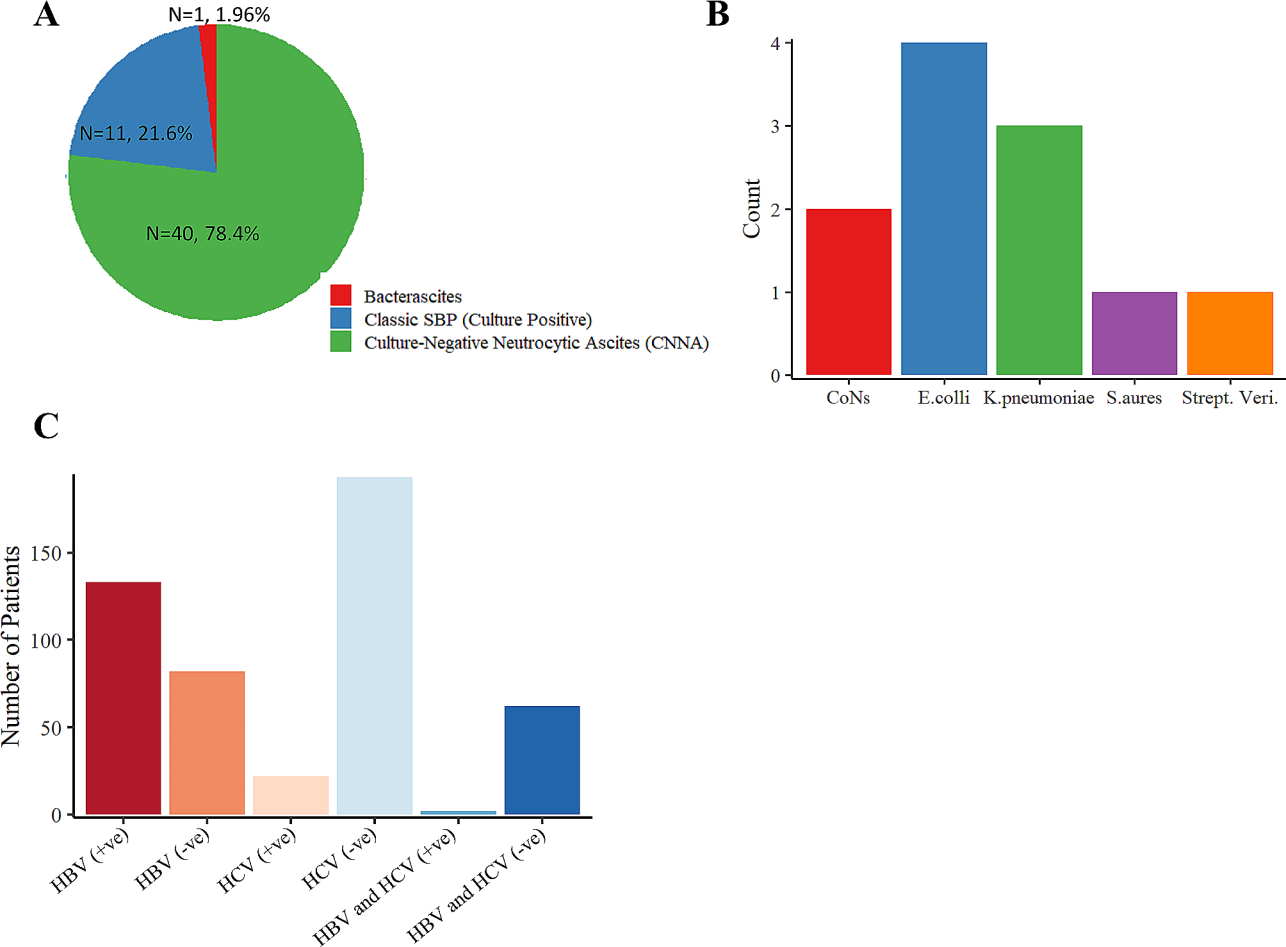
**- Prevalence of SPB, bacterial isolates, and seroprevalence of hepatitis Band C virus in Cirrhosis Patients. (A)**The prevalence of different types of spontaneous ascites fluid infection, **(B)** the different gram-positive and gram-negative bacterial isolates, **(C)** the Sero-positivity of HBV and HCV among study participants

SBP was present in 23.39% (51/218) -liver cirrhotic patients. Of the 51 patients who developed SBP, culture-positive SBP was present in 22% (11/50), and CNNA was found in 78.4% (40/50). The prevalence of MNB was 1.96% (1/51) in this study (Fig. 1a**)**. Among patients with culture-positive SBP, 4 (36.36%) isolates were *E. coli*, and 3 (27.27%) isolates were *Klebsiella* spp. Of the gram-positive bacteria, 1 (9.09%) isolate was a *Staphylococcus aureus*, 1 (9.09%) isolate was *Streptococcus viridian*, and 2 (18.18%) isolates were found to be CoNs (coagulase-negative staphylococcus species) (Fig. 1b**)**. We also assessed the prevalence of different hepatitis virus infections in these patients. Hepatitis markers were tested for 218 CLD patients, and about 61% (133/218) were positive for hepatitis B, 10.1% (22/218) were positive for hepatitis C virus, and 1% (2/218) were positive for hepatitis B and C virus, and 28.4% (62/218) were tested negative for both hepatitis B and C viruses (Fig. 1c).

### Antimicrobial-resistant profile of isolated bacterial isolates

We tested different antibiotics being employed to treat bacterial infections using the Kirby-Bauer disc diffusion method, as described in detail in the material and method part of this paper. The gram-negative isolate *K. pneumonia* showed 100% (3/3) sensitivity to Cefazolin, Cefepime, Cefotaxime, Cefotetan, Cefuroxime, Gentamicin, Meropenem, and Levofloxacin, whereas it showed 100% (3/3) resistance to amoxicillin/clavulanate (Table 4). On the other hand, *E. coli* showed 100% (4/4) sensitivity to Meropenem and Levofloxacin, whereas it showed 100% (4/4) resistance to amoxicillin/clavulanate, Ampicillin, and Cefazolin (Table 4). Both *K. pneumonia* and *E. coli* were 100% (7/7) resistant to amoxicillin/clavulanate and were 100% (7/7) sensitive to meropenem and levofloxacin (Table 4). From the gram-positive results, *S. aureus* was only resistant to penicillin, whereas *Str. viridian* was resistant to ceftriaxone, cefotaxime, cefepime, and penicillin. The isolated *S. aureus* was susceptible to Azitromycin, erythromycin, doxycycline, oxacillin, Gentamycin, and vancomycin, whereas *Str. viridian* was susceptible only to vancomycin *(*Table 4**).**

**Table 4:** Pattern of Drug resistance in bacterial isolate from cirrhosis patients

Gram Negative *N* (%)						
Antibiotics	*E.coli*			*K. pneumonia*		
	R	I	S	R	I	S
Amikacin	1 (25)	2 (50)	1 (25)		1 (33)	2 (67)
Amoxicillin/clavulanic acid	4 (100)			3 (100)		
Ampicillin	4 (100)			2 (67)	1(33)	
Cefazolin	4 (100)					3 (100)
Cefepime	2 (50)	1(25)	1 (25)			3 (100)
Cefotaxime	2 (50)		2 (50)			3 (100)
Cefotetan		1 (25)	3(75)			3 (100)
Cefoxitin	1 (25)	2 (50)	1 (25)	1 (33)		2 (67)
Ceftazidime	1 (25)	1 (25)	2 (50)	1 (33)		2 (67)
Ceftriaxone	2 (50)		2 (50)	2 (67)		1 (33)
Cefuroxime	2 (50)	1 (25)	1 (25)			3 (100)
Ciprofloxacin	1(25)	1 (25)	2 (50)		1 (33)	2 (67)
Gentamicin	3 (75)		1 (25)			3 (100)
Meropenem			4 (100)			3 (100)
Levofloxacin			4 (100)			3 (100)
Tobramycin	2 (50)	1 (25)	1 (25)		1 (33)	2 (67)
Trimethoprim\ sulfamethoxazole	3(75)	1 (25)			1 (33)	2 (67)
Gram Positive N (%)						
Antibiotics	*Streptococcus viridans*			*Staphylococcus aureus*		
	R	I	S	R	I	S
Ampicilin	1(100)					
Cefepime	1(100)					
Cefotaxime	1(100)					
Cefrtaxione	1 (100)					
Penicillin	1 (100)			1 (100)		
Vancomycin			1 (100)			1 (100)
Azithromycin						1 (100)
Clindamycin					1 (100)	
Erythromycin						1 (100)
Doxycycline						1 (100)
Oxacillin						1 (100)
Gentamycin						1 (100)
Sulfamethoxazole/ trimethoprim					1 (100)	
Tetracycline						1 (100)
Tobramycin					1 (100)	1 (100)

### Clinical and laboratory parameters were able to predict spontaneous bacterial peritonitis

A logistic regression was performed to appreciate the effect of age, gender, marital status, and clinical, and laboratory parameters on the likelihood of predicting SBP in cirrhosis patients. The logistic regression model showed that the variables age, gender, and marital status were statistically insignificant (*P* > 0.05) in predicting SBP. However, the clinical features of a history of jaundice, low arterial blood pressure on admission, and fever were found to be independent predictors (*P* < 0.05) of spontaneous bacterial peritonitis. Ultrasound studies depicted a shrunken livers and enlarged spleens, which were also found as independent predictors (*P* < 0.05) of spontaneous bacterial peritonitis (Table 5**).**

**Table 5:** Clinical predictors of SBP in cirrhosis patients

Independent Predictor	*p*-value	Adjusted odds ratio	95% CI
UGB	0.74	0.868	0.377–1.999
Abdominal Pain	0.598	1.259	0.534–2.968
Jaundice	0.004*	3.465	1.479–8.117
Sleeping disturbance	0.327	0.669	0.300–1.493
A previous episode of SBP	0.197	0.553	0.225–1.359
Abdominal distention	0.304	0.602	0.229–1.585
Weight Loss	0.223	1.752	0.711–4.317
Fever	0.032*	2.651	1.088–6.453
Chills	0.092	2.083	0.888–4.885
Pedal edema	0.498	1.383	0.541–3.531
Systolic blood pressure	0.0065*	4.794	1.552–14.809
Diastolic blood pressure	0.711	0.805	0.255–2.538
Body Mass Index	0.197	0.588	0.262–1.318
Maximum liver span	0.000*	7.521	2.620–21.590
Nodularity of liver surface (its longest axis)	0.313	1.577	0.650–3.822
Spleen size	0.023*	2.71	1.147–6.400
Collateral vessels	0.438	1.958	0.358–10.695
PVD	0.052	2.82	0.989–8.039

Similarly laboratory parameters were also able to significantly predict the presence of spontaneous bacterial peritonitis (SBP). Increased level of ALT (AOR = 1.02, *P* = 0.039, 95% CI (0.99–1.12), increased level of ALP (AOR = 1.05, *P* = 0.013, 95% CI (0.98–1.13), decreased level of serum albumin (AOR = 0.141, *P* = 0.03, 95% CI (0.034–0.58), increased number of total WBC count (AOR = 1.35, *P* = 0.0001, 95% CI (1.23–1.64)), and platlate count below 150,000/uL (OR = 0.67, *P* = 0.002, 95% CI (0.41–1.07)) were able to predict SBP (Table 6**)**.

**Table 6:** Laboratory predictors of SBP in cirrhosis patients

Independent Predictor	*P*-Value	Adjusted OR	95% CI OR
Ascites Fluid TP g/dL)	0.812	1.15	0.36–3.67
ALT(U/l)	0.039*	1.02	0.99–1.12
ALP(U/l)	0.013*	1.05	0.98–1.13
TB(U/l)	0.721	0.969	0.816–1.15
Serum Albumin (g/dL)	0.03	0.141	0.034 – 0.58
Creatinine (mg/dL)	0.344	0.74	0.41–1.39
Hepititis B	0.836	0.865	0.22–3.39
WBC (10^9^/L)	0.0001*	1.35	1.23–1.64
Hgb (g/dL)	0.353	0.905	0.733 – 1.12
PLT (10^3^/uL)	0.002*	0.67	0.41–1.07
Na (mmol/L)	0.76	1.02	0.89 – 1.15
K (mmol/L)	0.918	1.01	0.35 – 3.16
Cl (mmol/L)	0.586	0.865	0.986–1.01

## Discussion

Ascites is one of the most common complications in patients with cirrhosis [18]. One of the most common etiologies of cirrhosis has been related to hepatitis B infection, which is also common in our case [19]. Around 10–30% of patients with ascites develop spontaneous bacterial peritonitis (SBP), which is linked with high morbidity and mortality [20–22]. In our case, the prevalence of SBP was 23.34%, which is comparable to previous reports [23] and lower compared to a study conducted in Germany, which was 33.9%, and in Vietnam, which was 29.3% [24, 25]. Timely antimicrobial therapy includes a third-generation cephalosporin for community-acquired infection; nosocomial infections should be treated empirically with a carbapenem or with piperacillin-tazobactam, or based on local susceptibility testing. Patients with gastrointestinal (GI) hemorrhage should receive ceftriaxone prophylactically for GI hemorrhage [26]. Other studies have shown that follow-up of infected patients shows that 30% of patients die within 1 month after infection and another 30% die within 1 year [27]. Similarly, the odds (OR 2.522, 95% CI 1.044–6.091, *p* = 0.040) of mortality rate among cirrhosis patients acquiring infection during hospitalization are much higher compared to non-infected individuals [24]. This indicates that preventing infection in cirrhosis patients is crucial, as it decreases the likelihood of poor clinical outcomes in these patients. Early predictive parameters indicating infection among patients with cirrhosis could be important in preventing mortality in these patients.

On the other hand, the rate of culture-negative neutrocytic ascites was 78.43%, which is higher compared to previous reports, which reported around 27–60% [20, 28–30], and compared to other reports, which identified 64% of SBP as culture-negative [9]. Geographic factors and laboratory methods could contribute to this differences, but in our case and other reports, culture-negative neutrocytic ascites is the most common form of ascitic fluid infection. The most frequent bacterial isolate turned out to be gram-negative enteric bacteria, which is similar with other studies [31]. The common bacterial isolate was *E. coli* (36.3%, *n* = 4), which agrees with other reports [7, 32–34]. Of the gram-positive bacterial isolates, coagulase-negative staphylococcus species were common, accounting for 18.18% and aligning with other studies [9]. Although the frequency of the bacterial isolates from our study was small, we observed a pattern of resistance. The isolated gram-negative bacteria showed a full resistance to amoxicillin/clavulanic [35].

However, isolated gram-positive bacteria showed resistance to penicillin. The outcome of patients with SBP is poor since chronic liver failure and death occur in 60% and 40% of the patients, respectively, and early antibiotic treatment for these patients is crucial. However, the increase in microbiological resistance makes current management more challenging [36]. We found that antibiotics like *Meropenem* and *levofloxacin* were effective against gram-negative bacteria, and *vancomycin* was effective against gram-positive bacteria and can be used in treating SBP patients. As the standard treatment for SBP mainly depends on prompt broad-spectrum antibiotic administration, isolating the causative agents and profiling the resistance pattern in different areas is important [37].

One of the most important findings of this study was the role of clinical and laboratory parameters as predictors of SBP. We found that clinical data like maximum liver span, spleen size, fever, and jaundice were important predictors of SBP. For instance, other studies showed that fever was one of the predictors of SBP in children with cirrhosis [38]. Likewise, laboratory parameters including platelet count, WBC count, and ascitic fluid levels of albumin, were important predictors of SBP in cirrhosis patients. Studies showed that platelet count predict SBP [39, 40], which is complimentary with our finding, whereas others found age, sex, diabetes [41], the value of INR [7] and neutrophil-to-lymphocyte ratio (NLR) [42] as positive predictors of SBP. In other studies, the presence of inflammatory markers like IL-6 in the blood was correlated with disease severity in patients with ascites [43]. Likewise, simple immunological measurements like lymphocyte-to-monocyte ratio were found to be the best predictors of bacterial infection in patients with liver cirrhosis [44]. Overall, the measured parameters in our study and others can be easily performed, used as a simple indicator of SBP, and help to initiate early medical intervention.

To conclude, as the mortality rate after SBP is high [22], prompt diagnosis and treatment, understanding the microbial agent, resistant profile, and predictive parameters in different settings are crucial. Here, we conducted the first study in Ethiopia on ascites infections among cirrhosis patients that have developed ascites and found that ascites fluid infection is common in these patients. The combination of clinical data and laboratory parameters can be used for rapid diagnosis or exclusion of SBP and to initiate evidence-based treatment for these patients. Furthermore, the most common form of SBP was culture-negative neutrocytic ascites, and even if the amount of bacterial isolate was low, a pattern of drug resistance was still evident. One limitation of this study was that the bacterial isolates were too small to fully elucidate the pattern of drug resistance among the isolated pathogens; thus, we suggest further study in other hospitals focusing on culture-positive SBP to further strengthen our finding.

### Electronic supplementary material

Below is the link to the electronic supplementary material.

Supplementary Material 1

## Data Availability

All data supporting the findings of this study are available within the paper, incorporated in table form and the attached its Supplementary Information.

## Abbreviations

ALP: Alkaline phosphatase
ALT: Alanine aminotransferine
ATCC: American Type Culture Collection
CLD: Chronic Liver Disease
CLSI: Clinical Laboratory Standardization Institute
CNNA: Culture negative neutrocytic ascites
CoNS: Coagulase negative Staphylococus
HBV: Hepatitis B Virus
HCV: Hepatitis C Virus
INR: International Normalization ratio
NLR: Neutrophil to lymphocyte ratio
NNBA: Non neutrocytic bacteriascites
SAFI: Spontaneous ascites fluid infection
SBP: Spontaneous bacterial infection
SPSS: Statistical package for social science
